# *Litomosoides* sp. (Filarioidea: Onchocercidae) Infection in Frugivorous Bats (*Artibeus* spp.): Pathological Features, Molecular Evidence, and Prevalence

**DOI:** 10.3390/tropicalmed4020077

**Published:** 2019-05-10

**Authors:** Emilio Rendón-Franco, Osvaldo López-Díaz, Fernando Martínez-Hernández, Guiehdani Villalobos, Claudia Irais Muñoz-García, Nidia Aréchiga-Ceballos, Jorge Alberto Alfonso-Toledo, María Martha García Flores, Alvaro Aguilar Setién

**Affiliations:** 1 Departamento de Producción Agrícola y Animal, Universidad Autónoma Metropolitana Unidad Xochimilco, Calzada del Hueso 1100, Col. Villa Quietud, Del. Coyoacán, Ciudad de México CP. 04960, Mexico; emilio.rendon.franco@gmail.com (E.R.-F.); voxis@hotmail.com (O.L.-D.); clau_irais_munoz@hotmail.com (C.I.M.-G.); 2 Departamento de Ecología de Agentes Patógenos, Hospital General “Dr. Manuel Gea González”, Calzada de Tlalpan # 4800, Del. Tlalpan, Ciudad de México CP. 14080, Mexico; fherxyz@yahoo.com (F.M.-H.); guiehda@yahoo.com.mx (G.V.); 3 Laboratorio de Rabia, Instituto de Diagnóstico y Referencia Epidemiológicos, Francisco de P. Miranda 177, Colonia Unidad Lomas de Plateros, Ciudad de México CP. 01480, Mexico; nidia.arechiga@salud.gob.mx; 4 Unidad de Investigación Médica en Inmunología, Coordinación de Investigación Médica, Instituto Mexicano del Seguro Social (IMSS), Av. Cuauhtémoc No. 330, Col. Doctores, UMAE Hospital de Pediatría, C.M.N. Siglo XXI, Ciudad de México CP. 06720, Mexico; ja.alfonsotoledo@gmail.com (J.A.A.-T.); mmgarcia_3001@yahoo.com.mx (M.M.G.F.); 5 Facultad de Medicina Veterinaria y Zootecnia, Universidad Nacional Autónoma de México, Circuito Exterior s/n, Ciudad Universitaria, Del. Coyoacán, Ciudad de México CP. 04510, Mexico

**Keywords:** parasite, microfilariae, frugivorous bats

## Abstract

Bats can host pathogenic organisms such as viruses and fungi, but little is known about the pathogenicity of their parasites. Hemoparasites are frequently recorded in Neotropical bats, particularly *Litomosoides* (Filarioidea: Onchocercidae), but their pathogenic effect on bats is scarcely known. In this work, *Litomosoides* microfilariae were identified in four (8%) out of 51 sampled frugivorous bats belonging to three different species: *Artibeus aztecus*, *Artibeus jamaicensis*, and *Artibeus lituratus*, which are located in Yautepec, Morelos, Mexico. Two infected animals showed weakness, tachypnoea, and ecchymosis on their wings. In these animals, histopathology revealed microfilariae in the blood vessels of the lung, liver, and spleen. Both animals presented exudative pneumonia with congestion and concomitant edema, in addition to moderate arterial hypertrophy. Parasitemia was quantified in blood samples of the infected animals (>3000 parasites/mL). Phylogenetic analysis placed the obtained sequence inside the *Litomosoides* genus, reaching over 98% identity to the related species. Due to the relevance of bats in ecosystems, any new record of their parasite repertoire offers noteworthy insights into our understanding of the ecology and impact of new parasite species in bats.

## 1. Introduction

Bats are currently recognized for hosting a large biodiversity of microorganisms that can affect humans [1] or their own health [2]. It has been established that these animals are reservoirs for important emergent diseases such as the Marburg virus, SARS, MERS, and others. Some studies have shown that *Lyssaviruses* (the viral genus where *Rabies virus* belongs) evolved first in the order Chiroptera and then spread to other mammals [3]. Recently, many studies have focused on *Pseudogymnoascus destructans*, an emergent lethal fungus that has killed millions of North American bats [2]. However, few studies have focused on the relationship between parasites and bats, and little is known about their pathology [4]. The case of hemoparasites is interesting as they are frequently recorded in Neotropical bats, particularly in filarial nematodes (Onchocercidae) of the genus *Litomosoides* [5]. However, although tissue pathologies caused by filarial nematodes are known in wild mammals [6], there have been no records in bats. Additionally, molecular detection for bat filarial parasites has never been used. 

Filarioid nematodes of the genus *Litomosoides* occur in the thoracic and (or) abdominal cavities of marsupials, rodents, and bats of the Nearctic and Neotropical regions. This genus includes at least 31 species [7]. Some authors have proposed that *Litomosoides* evolved as a bat parasite and later diversified in rodents [8,9], but others have postulated the opposite [10]. The most studied species is *L. sigmodontis* because it is one of the best murine models for human filariasis. Additionally, the species also shares many migration patterns as well as biochemical and genetic features with the filarial worms *Brugia* spp., *Wuchereria bancrofti*, *Loa loa*, and *Onchocerca* spp. [11]. The *Litomosoides* life cycle includes an invertebrate host, usually dermanyssoid mites [12]. Like in other filarial nematodes, their life cycle stages include four larval instars before the adult stage. After the ingestion of first-stage larvae by the intermediate host, the nematode develops up to third-stage larvae, which migrate to the arthropod’s mouthparts. When the vector feeds on blood, stage III larvae are inoculated and develop to the migrating stage IV larvae and finally into the adult worm, which is located mainly in the pleural cavity. Adult worms mate and the microfilariae (Mf) released by females enter the host´s lymphatic system and blood vessels [11]. 

Unfortunately, *Litomosoides* species other than *L. sigmodontis* remain under studied, especially those isolated from bats. These bat filarids seem to preferentially target the phyllostomid group of the genus *Artibeus* [5,7]. This genus is composed by frugivorous bats, which are abundant and widely distributed in the Neotropical area from Mexico to Argentina. Since their main role in the ecosystem is seed dispersal, they are involved in forest regeneration and the maintenance of floral diversity [13]. This study aimed to identify *Litomosoides* infection in Mexican *Artibeus* bats by morphological and molecular methods, and to explore some of the pathological alterations produced by this parasite. 

## 2. Materials and Methods

### 2.1. Study Area and Animals Capture

Part of an ongoing project aimed at determining the susceptibility of bats to Zika virus, this study was carried out in the municipality of Yautepec, located in the State of Morelos, Mexico (18°54′23″ N, 98°58′13″ W). The project was carried out under the authorization of the Instituto Mexicano del Seguro Social, permit reference: protocol IMSS number R-2017-785-092 CNIC, and of the Secretaría de Medio Ambiente y Recursos Naturales, permit reference: SEMARNAT 09/K5-1345/08/18. 

Bats were captured during two periods: on March and September of 2018, using two 12-m mist nets during two consecutive nights. Bat species were identified according to Medellin et al. taxonomic key [14]. 

### 2.2. Collection of Biological Material

During the first capture for all specimens, we gathered information of their health status and reproductive condition. Samples of blood, urine, feces, and ectoparasites were also taken from all captured animals as part of the clinical examination. Blood samples were taken by the puncture of the marginal vein of the wing, and the whole blood was collected into EDTA vacutainer microtubes^®^. From each animal, thin blood smears were made and immediately fixed and stained with a 1:1 solution of 95 mL of 5% formalin, 5 mL acetic acid, and 2 mL saturated alcoholic crystal violet solution. 

Two adult male bats of the species *A. aztecus* were trapped. While they were handled, some clinical signs such as weakness, tachypnoea, and ecchymosis on the wings were noted. Due to the severity of the signs, euthanasia was performed, followed by immediate necropsies. For histopathological analysis, samples were taken from the skin, spleen, kidney, lung, and liver tissues and then fixed in 10% formalin. Spleens were frozen at −40 °C for DNA extraction. 

During the second capture, all trapped bats were sampled for blood, which was treated as described previously. After blood collection, all bats were released. During this capture, none of the bats showed apparent clinical signs. 

### 2.3. Microfilaria Identification and Parasite Load Calculation

All Mf detected in blood smears were examined under the microscope at 400× and 1000× magnification. Only stretched Mf were used for morphological analysis, and the following characteristics were measured: total body length and body width at the nerve ring. Additionally, the position and shape of the terminal nucleus were checked. Mf were identified using the taxonomic keys of Esslinger (1973) [15], Anderson and Bain (1976) [16], Chabaud and Bain (1976) [17], and Guerrero et al. (2002) [7]. 

When blood samples with a volume of at least 250 µL were obtained from Mf-positive animals, parasites were counted. Parasite load (the average number of parasites per mL) was calculated as the average of five counts in a Neubauer’s chamber. 

### 2.4. Molecular DNA Analysis

Fragments of fresh spleen from the euthanized animals were homogenized, and DNA was extracted using a phenol-chloroform technique [18]. PCR was performed to amplify a fragment of the small subunit ribosomal DNA (SSUrDNA) region. Primers targeted the highly conserved sequences reported in GenBank and included the forward primer 5′-CCG CTT TTC TCG AAA CGG CTC A-3′ and the reverse primer 3′-GAC GGG CAG CTT CCG GAA ACG-5′ [6]. PCR amplifications were carried out in a final volume of 25 μL, containing 200 ng of DNA as the template, 20 pmol of each primer, 1 × PCR buffer (8 mM Tris–HCl, pH 8, 20 mM KCl), 1.5 mM MgCl_2_, 0.5 mM dNTPs, 1 μL of BSA (1%), and 2 U of Taq DNA Polymerase (Promega, Madison, WI, USA). Amplification conditions were one cycle at 94 °C for 5 min; 35 cycles including denaturation, annealing, and extension steps at 94 °C for 30 s, 60 °C for 1 min, and 72 °C for 30 s, respectively; and a final extension step at 72 °C for 7 min. The presence of amplicons was confirmed by electrophoresis in 1.5% agarose gels, after which the band was purified using the AxyPrep PCR clean-up kit (Axigen Biosciences, CA, USA) and sequenced on both strands by a commercial supplier. The sequence of 529 bp was deposited in GenBank (MK458934). 

The phylogenetic analysis was set up with all SSUrDNA sequences of the Filarioidea family available in GenBank and those obtained in this study. Multiples alignments with MEGA 5.01 were undertaken; the molecular evolution model to build the phylogenetic tree was the General Time Reversible model with Gamma distribution and invariable sites (GTR + G + I), determined using the JModeltest 3.7 software [19]. Phylogenetic analysis was performed using a Bayesian model in Mr. Bayes software version 3.01 [20]. 

### 2.5. Prevalence Analysis

Following the finding of the filarial parasites during the first capture, the second capture was performed in order to establish filarial prevalence in the *Artibeus* bats of the area. The prevalence was defined as the number of individuals with at least one filarial parasite divided by the number of individuals examined in both captures. 

### 2.6. Histopathological Analysis

Formalin-fixed tissues (skin, spleen, kidney, lung, and liver) from *A. aztecus*, collected during the first capture, were embedded in paraffin. Tissue blocks were prepared, sectioned with a microtome at a thickness of 5 μm, and stained with hematoxylin and eosin. 

## 3. Results

Blood smears from four out of the 51 individuals analyzed (10 *A. aztecus*, 24 *A. jamaicensis*, 13 *A. lituratus,* and four *Sturnira lilum*) were positive for microfilariae. Of the positive animals, two were *A. aztecus* (both were euthanized) from the first capture period, and the other two were *A. jamaicensis* and *A. lituratus* from the second period. 

### 3.1. Filariae Identification

**Figure 1:**
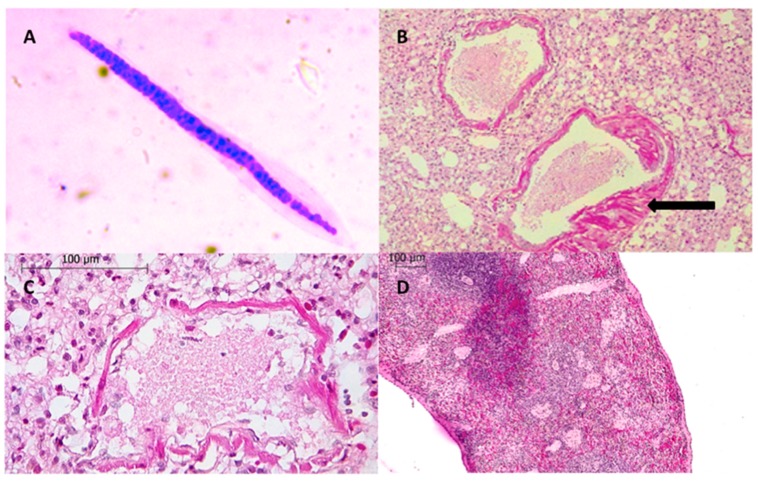
(**A**) Blood smear. Sheathed microfilaria of *Litomosoides* sp. (**B**) Pulmonary arterial vessel. The black arrow indicates smooth-muscle hypertrophy. (**C**) Exudative pneumonia with an inflammatory infiltrate composed predominantly of neutrophils, the tissue also displayed congestion. (**D**) Spleen. Moderate lymphoid depletion and mild lymphoid hyperplasia.

Morphological characteristics of Mf in the four positive animals were similar and consistent with Litomosoides [7,15,16,17]. All Mf were folded, and their sheaths were constricted at the first two-thirds of the body to form a distal globular shape. Total length ranged from 69 to 85 μm, body width at the nerve ring ranged from 2.9 to 5.0 μm, and tails were not curved (Figure 1A). Table 1 shows the detailed Mf measurements for the bat species. Litomosoides adult stages were not detected in any of the two *A. aztecus*. 

**Figure 2:**
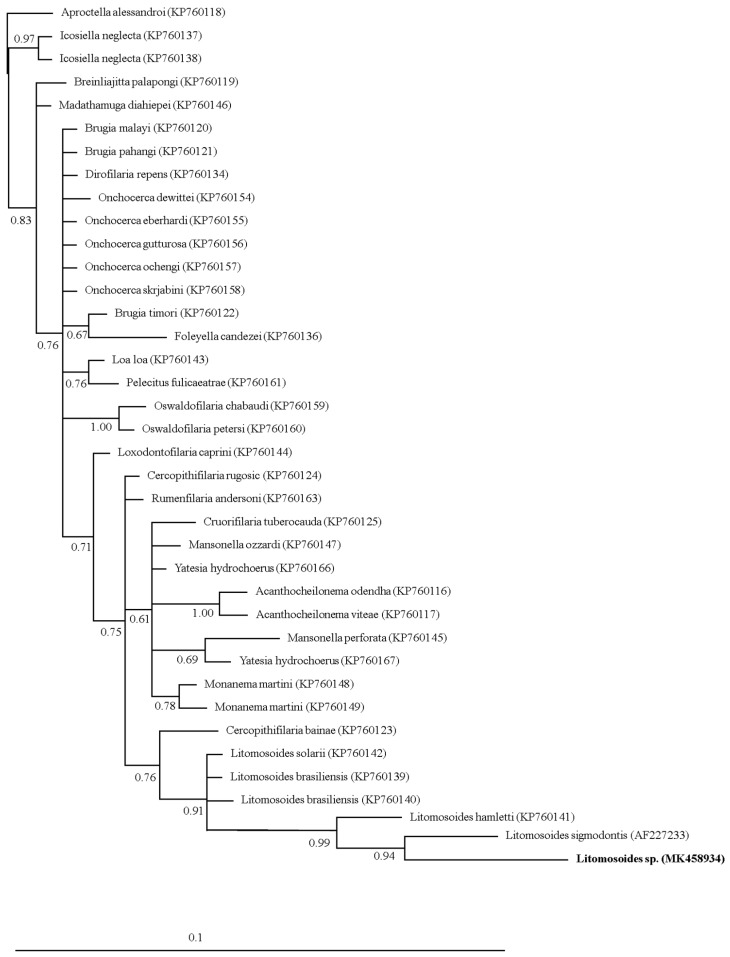
Bayesian phylogenetic tree using the SSUrDNA sequences from different species of filarial parasites. The sequence obtained in this work is shown in bold. The numbers of the nodes indicate the values of the support or posterior probability.

The sequence obtained from the SSUrDNA gene identified Mf as *Litomosoides* sp. (Figure 2). The sequence showed a 98.6% (7/529) identity with *Litomosoides sigmodontis* and 98.1% to 98.5% identities with the other available sequences of *Litomosoides* spp. (*L. brasiliensis*, *L. hamletii*, and *L. solaris*). Phylogenetic reconstruction performed with Bayesian analysis using Filarioidea family sequences available in GenBank showed that the sequence obtained in this study fitted within the Litomosoides species clade, with its closest relative being *L. sigmodontis* (0.94 of posterior probability). 

### 3.2. Parasite Load

The parasitic loads were as follows (Mf mean ± SD): the two *A. aztecus* had 3950 ± 255 and 3850 ± 348, and the *A. jamaicensis* had 3287 ± 353. 

### 3.3. Histopathology

**Figure 3:**
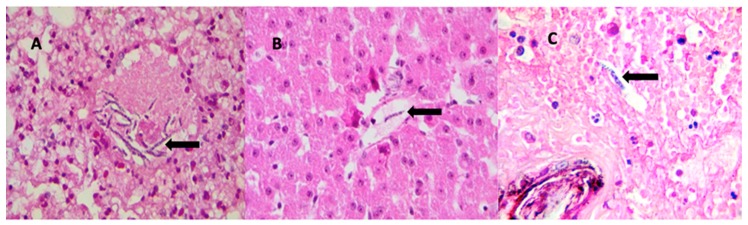
Mf in blood vessels of (**A**) lung, (**B**) liver, and (**C**) skin. Black arrows indicate microfilariae.

Mf were observed in the skin, liver, and into the lumen of pulmonary and splenic blood vessels (Figure 3). Of the four parenchymatous tissues collected, the only one without Mf was the kidney. The most relevant finding in the lung was a large number of Mf in the blood vessels. Lesions detected in the lungs included moderate multifocal exudative pneumonia with an inflammatory infiltrate composed predominantly of neutrophils, plus congestion and concurrent moderate multifocal edema. Moderate arterial smooth-muscle hypertrophy was also detected (Figure 1B,C). Splenic sections revealed moderate and diffuse lymphoid hyperplasia and lymphoid depletion (Figure 1D). 

## 4. Discussion

This study reports, for the first time, pathological lesions in bats associated with *Litomosoides* Mf. Additionally, this is the first report of *Litomosoides* prevalence and parasitic loads in a phyllostomid bat community from Mexico. Finally, this is the first published *Litomosoides* sequence from a bat host. As Muñoz-García et al. (2018) mentioned in [6], adding new filarid sequences should help improve diagnosis. Genetic analyses using the SSUrDNA marker showed scarce genetic variation between Filarioidea species. However, in the phylogenetic reconstruction, the *Litomosoides* spp. clade that encompassed our sequence showed a high posterior probability value (0.91). The sequence obtained showed the highest similarity to *L. sigmodontis* and a high identity with *L. hamletti L. brasilensis*, the latter of which has been a species reported in Mexico [10]. Although the sequence obtained in this study was clustered in the *Litomosoides* clade, SSUrDNA markers were highly conserved. It is thus unclear whether the filarial species reported in this work corresponds to a new or an existing species of *Litomosoides*. More morphological studies accompanied by its molecular markers are hence necessary. 

Since *Litomosoides* spp. infections in Neotropical bats have been frequently reported [5], it is surprising that no one has ever attempted to identify the effect of this parasite on bats. Conversely, in murine models, pathological effects have been well described [21]. A study in cotton rats (*Sigmodon hispidus*), naturally infected with *L. sigmodontis*, found histological characteristics different from those observed in this study. For example, no damage in blood vessels was found, even when some of the vessels carried heavy feature loads of Mf [22]. In that study, pneumonia was not reported as a characteristic of *Litomosoides* infection. Nevertheless, similar lesions and pathologic findings to those observed in the present study have been previously described in studies that looked at naturally infected *S. hispidus* and additional murine models (*Rattus rattus* and *Mastomys natalensis*) experimentally infected with *L. carinii*. These reported pathological changes such as edema, congestion, and neutrophil infiltration in the lungs as well as lymphoid depletion of the spleen [22,23,24]. On the other hand, smooth-muscle hypertrophy of pulmonary arteries has not been previously associated with *Litomosoides* infection. This lesion could resemble a chronic process that has been reported in dogs parasitized with *Dirofilaria immitis* [25,26]. Unlike in our study where the most common vascular lesion was hypertrophy of the medial layer, in most studies in dogs with dirofilariosis, the most common were hyperplasia and hypertrophy of the capillary endothelium [25,26,27]. In a murine model infected with *L. sigmodontis*, Mf presence inside blood vessels of the lungs was proposed as an anatomic reservoir site during the patency of the disease [21]. The same study further proposed the said vessels as sites for larvae elimination, in addition to the spleen and liver [21]. This potentially reinforces the hypothesis of a chronic infection in both *A. aztecus* bats, since Mf were found in all three organs. 

Lesions detected in bats were not clearly associated with filarial infection. Filarial antigens could activate different pathways of the immune response, as shown by the histological changes reported by Zahner et al. (1987) [24] and Tarish and Atwell (1989) [26]. In these studies, tissue alterations were induced by the sole inoculation of the *L. carinii*-adult phase antigen in *M. natalensis* and of *D. immitis* in dogs, respectively. This suggests that filarial lesions can be caused by a cellular immune response toward circulating molecules (antigens). On the other hand, lymphoid depletion implies immunosuppression. This process has been logically associated with *Litomosoides* spp. through the recruitment of CD4 + Foxp3 + Treg cells [28]. 

In addition to the histopathological changes, clinical signs and blood-circulating Mf loads (more 3 × 10^3^ Mf/mL) were also recorded in the two infected *A. aztecus*. These animals showed signs of weakness, tachypnoea, and hemorrhage, likely related to parasitemia-induced pathologies. Cottontail et al. (2009) found, in 40 *A. jamaicensis* infected with *Litomosoides* spp., a range from 0 to 278 × 10^3^ Mf/mL, with a mean of 3.6 × 10^3^ Mf/mL [29], a close value to the means reported in our study. In a rat model (*M. natalensis*) infected with *L. carinii*, parasitemia ranged from 3 to 623 × 10^3^ Mf/mL. The highest and lowest loads happened at 150 and 400 days postinfection, respectively [24]. Interestingly, the lowest load (3 × 10^3^ Mf/mL) corresponded to the end of the disease course, which reinforces our chronic hypothesis for both *A. aztecus* bats. Zahner et al. (1987) [24] found that when microfilaremia was at its lowest level (3 × 10^3^ Mf/mL), only a few fragments of adult nematodes were found in which there was a partial match to our findings in both *A. aztecus* where no adults were found. This could be explained by a degradation process that occurs during the chronic phase of disease. It is important to consider that natural infections are usually caused by smaller parasitic loads than experimental ones, thereby reducing the chances of finding adult nematodes. Therefore, it is important to continue detailing reporting the findings of natural infections. 

In former *Litomosoides* studies undertaken in bats, other researchers did not perform histological analysis or report any clinical signs. In the study performed by Cottontail et al. (2009) [29], they performed blood cell counts and showed changes in leukograms associated with *Litomosoides* infection. The alterations were characterized by eosinophilia and increased immature neutrophils highlighting a cellular response [29]. In murine models infected with *Litomosoides*, it is well known that Mf presence induces eosinophil recruitment in blood and lungs [21]. Unfortunately in the present study, leukograms were not performed. Such findings are uncommon in animals infected with *Litomosoides*. In contrast, pronounced inflammation characterized by neutrophil infiltration has been reported for other filarial-related pathologies such as onchocerca skin abscesses and ocular onchocerciasis [30,31]. Interestingly, a murine model for ocular onchocerciasis caused by *Onchocerca volvulus* revealed that it was *Wolbachia* (a symbiotic bacterium) and not the parasite that induced a cellular response mediated by neutrophils, the results suggesting an essential role of *Wolbachia* in filarial pathogenesis [31]. 

Prevalence in our study (8%) was similar to that reported for *A. jamaicensis* in some localities in Panama. Other bat species reported in that study showed higher loads than the ones we found [29]. The authors did not find any associations between environmental variables, biodiversity, and prevalence. *Artibeus* bats are common and well-studied, possibly skewing the prevalence of these parasites in this bat species. 

Frugivorous bats are important for the ecosystem because they spread the seeds of fruit trees, particularly in tropical and sub-tropical environments [32]. Bats that are parasitized by *Litomosoides* can show weakness, which in turn may lower their efficiency in spreading seeds. These animals may also be more prone to acquire and spillover other pathogenic microorganisms. Due to the relevance these bats have in ecosystems, any new record of their parasite repertoire offers noteworthy insights into our understanding of their ecology and environmental impact. 

## Figures and Tables

**Table 1:** Microfilariae morphometric per bat host species.

Host Species	Prevalence % (Positive Bats/Total Bats)	Number of Microfilariae Tested	Body Length (µm), Mean ± SD	Body Width at the Nerve Ring (µm), Mean ± SD
*Artibeus aztecus*	20 (2/10)	8	74.9 (2.9)	3.3 (0.4)
*Artibeus jamaicensis*	4.2 (1/24)	4	77.6 (6.2)	4.3 (0.5)
*Artibeus lituratus*	7.7 (1/13)	4	78.8 (4.8)	4.8 (2.5)
